# Initial presentations and final outcomes of primary pyogenic liver abscess: a cross-sectional study

**DOI:** 10.1186/1471-230X-14-133

**Published:** 2014-07-28

**Authors:** Chang-Hua Chen, Shung-Sheng Wu, Hung-Chi Chang, Yu-Jun Chang

**Affiliations:** 1 Division of Infectious Diseases, Department of Internal Medicine, Changhua Christian Hospital, 135 Nanxiao St., Changhua City, Changhua County 500, Taiwan; 2 Division of Gastroenterology, Department of Internal Medicine, Changhua Christian Hospital, 135 Nanxiao St., Changhua City, Changhua County 500, Taiwan; 3 Department of Surgery, Changhua Christian Hospital, 135 Nanxiao St., Changhua City, Changhua County 500, Taiwan; 4 Epidemiology and Biostatistics Center, Changhua Christian Hospital, 135 Nanxiao St., Changhua City, Changhua County 500, Taiwan; 5 Department of Nursing, College of Medicine & Nursing, Hung Kuang University, No. 1018, Sec. 6, Taiwan Boulevard, Shalu District, Taichung 43302, Taiwan

**Keywords:** Primary pyogenic liver abscess, Initial presentation, Outcome complications, Mortality, Risk factors

## Abstract

**Background:**

Although pyogenic liver abscess (PPLA) fatalities are decreasing owing to early diagnosis and effective treatments, PPLA-associated complications still exist. The purpose of this study was to analyze the characteristic features of initial presentations and final outcomes of PPLA caused by different pathogens.

**Methods:**

This retrospective study collected and analyzed information regarding initial presentations and final outcomes in patients diagnosed with PPLA at admitted at Changhua Christian Hospital from January 1 to December 31, 2010.

**Results:**

During the study period, we analyzed the records of a total of 134 patients with documented PPLA. There were no significant causative pathogen-related differences in symptoms at initial presentation. Compared with the survivor group, patients in the mortality group were characterized by male gender (*p* < 0.001), malignancy (*p* < 0.001), respiratory distress (*p* =0.007), low blood pressure (*p* = 0.024), jaundice (*p* = < 0.001), rupture of liver abscess (*p* < 0.001), endophthalmitis (*p* = 0.003), and multiple organ failure (*p* < 0.001). No patients received liver transplantation or were diagnosed with HIV during the study period. According to univariate logistic regression analysis, gender (OR = 1.185, 95% CI: 0.284–11.130, *p* = 0.006), malignancy (OR = 2.067, 95% CI: 1.174–13.130, *p* = 0.004), respiratory distress (OR = 1.667, 95% CI: 1.164–14.210, *p* = 0.006), low blood pressure (OR = 2.167, 95% CI: 2.104–13.150, *p* = 0.003), jaundice (OR = 1.9, 95% CI: 1.246–3.297, *p* = 0.008), rupture of liver abscess (OR = 5.167, 95% CI: 2.194–23.150, *p* = 0.003), endophthalmitis (OR = 2.167, 95% CI: 1.234–13.140, *p* = 0.005), and multiple organ failure (OR = 3.067, 95% CI: 1.184–15.150, *p* = 0.001) differed significantly between the mortality and survivor groups.

**Conclusion:**

Although the initial presentations of PPLA caused by different pathogens were similar, there were significant differences in mortality in cases involving: (1) male patients, (2) malignancy, (3) initial respiratory distress, (4) initial low blood pressure, (5) jaundice, (6) rupture of liver abscess, (7) endophthalmitis, , and (8) multiple organ failure. We strongly recommend using a severity score of the disease to determine the risk of mortality for each patient with PPLA. In order to prevent complications and reduce mortality, more attention must be paid to high-risk PPLA patients.

## Background

Primary pyogenic liver abscess (PPLA) is not a rare disease in Taiwan [1]. With the introduction of percutaneous drainage, surgical drainage, and effective antibiotics, the treatment of PPLA has improved over the last 10 years [2]. However, PPLA still causes significant morbidity and mortality and if not diagnosed and treated early, is almost always fatal [3].

The clinical presentation of liver abscess in Taiwan has changed over the years owing to antibiotic overuse and associated drug resistance, higher prevalence of chronic or malignant diseases, and population ageing [4]. Despite the increased understanding of this new invasive syndrome [4], only earlier diagnosis and effective treatment can minimize the occurrence of sequelae and improve clinical outcomes. However, the literature describing initial clinical presentations of PPLA and early ultrasonography findings is sparse [5].

Since the clinical symptoms of PPLA are variable, and some of the PPLA causative pathogens, such as *Klebsiella pneumoniae* (*K. pneumoniae*), are highly resistant to the most effective antibiotic therapies, it is very important to develop accurate and effective diagnosis and treatment approaches for PPLA. Early diagnosis of patients with liver abscess is one of the most challenging tasks for a physician. Over the past 3 decades, ultrasound has had an important role in the diagnosis of this disease. Even with aggressive treatment with antibiotics and drainage, however, the mortality rate of liver abscess remains high [6-8].

It is often difficult to promptly diagnose the initial stage of PPLA because ultrasound findings are typically negative or non-specific and subclinical symptoms and signs are absent. We performed a cross-sectional study of all of the confirmed cases of PPLA in central Taiwan in order to (1) elucidate the risk factors for PPLA, (2) compare the most common ultrasound findings between PPLA cases caused by different pathogens, and (3) identify the prognostic factors for mortality.

## Methods

### Setting and population

The population of Changhua is mostly served by the Changhua Christian Hospital System (CCHS), which has maintained comprehensive clinical records since 1867. Changhua County is located in central Taiwan, and it had a population of 140,277 in 2000. Changhua Christian Hospital (CCH), a 1,800-beds tertiary referral medical center located in northern Changhua County, is a component of CCHS. CCH has been a Joint Commission International-accredited hospital since 2009. This cross-sectional retrospective study of PPLA was conducted by reviewing the computerized medical records collected at CCH from January 1 to December 31, 2010. The study was approved by the institutional review board of Changhua Christian Hospital (CCH IRB No. 131116).

### Case findings and analysis

Cases of PPLA at CCH were identified using the microbiological databases and medical records in the International Classification of Diseases, Ninth revision, Clinical Modifications (ICD-9-CM). We used ICD-9-CM code number 572.0 to find the cases. Each case had a medical chart that contained medical diagnoses, surgical interventions, and other key data from the medical records. The medical records of all the PPLA cases were manually reviewed by the primary investigator (C.C.H.) to confirm the diagnosis (using CCHS resources). Cases that were judged ambiguous were reviewed by the secondary investigator (S.S.W.). For example, although in some patients the abscess culture revealed *Corynebacterium urealyticum*, blood cultures grew *Staphylococcus epidermidis*, possibly because of skin contamination. Discrepancies between the abscess and blood cultures were also observed in some cases with secondary pyogenic liver abscess, such as after foreign body penetration of the alimentary tract. Only the first episode of PPLA in each person during the study period was included in statistical analyses.

PPLA was diagnosed based on clinical presentation, imaging findings of ultrasonography, abdominal computed tomography (CT) scans, and microbiological results. The treatment protocol for PPLA is described in the Additional file 1. The choice of the antimicrobial agent for the treatment was based on the results of the antibiotic susceptibility test. The following patient details were recorded: age, sex, date of admission, severity of underlying disease, comorbidity, diagnosis, clinical signs and symptoms, microbiological results, response to therapy, complications, and outcome. Only primary cases were collected, whereas those cases with distant metastasis from significant pyogenic infectious sources other than the liver were excluded. The severity of underlying disease was categorized according to the McCabe & Jackson criteria [9]. Microbiological data were collected only at the initial stage before the antibiotic therapy. All bacterial pathogens were collected, including anaerobes. If both blood and pus cultures were positive, we analyzed only the pus culture taken directly from the liver abscess. If there were recurrent PPLA episodes, only the first instance was taken into account.

Microbiological data were collected by reviewing the computerized records of the Microbiological Laboratory of CCH dated from January 1 to December 31, 2010. Blood culture analysis was performed for every case with suspected sepsis. We used a BACTEC NR-860 system (Becton Dickinson Diagnostic Instrument Systems, Cockeysville, MD, USA) to detect pathogens, Gram staining to determine morphology, and the API-20 series kit (bioMerieux Vitek, Hazelwood, MO) for biochemical reactions. Antibiotic susceptibilities were determined using the disk diffusion method (BBL™ Sensi-Disc™, Becton Dickinson) according to the guidelines of the Clinical and Laboratory Standards Institute [10].

### Definitions

PPLA was defined as the final diagnosis of primary pyogenic liver abscess in an adult patient (age ≥18 years) confirmed with clinical presentation, imaging findings, microbiological results, and/or pathological findings. Patients with the following characteristics were excluded: (1) age <18 years; (2) unavailable blood and pus culture data; (3) incomplete data; (4) inconsistencies among ICD-9-CM data, microbiological data, and medical records; (5) amebic liver abscess; and (6) secondary pyogenic liver abscess, such as pyogenic liver abscess secondary to foreign body penetration of the alimentary tract. Initial presentation was defined as the symptoms, signs, laboratory data (including microbiological results), and image findings observed within 48 h of admission. Patient delay was defined as the time interval between the onset of symptoms and presentation to the hospital. Delay of antibiotic therapy was defined as the time interval between the presentation to the hospital and the start of antibiotic therapy. Prolonged delay was defined as a delay greater than the median. We defined final outcome as either a status with complications or patient’s death. PPLA complications were rupture of liver abscess, endophthalmitis, and multiple (more than 3) organ failures attributable to PPLA. The *K. pneumoniae* group included patients whose microbiological culture revealed *K. pneumoniae*. The non-*Klebsiella* Gram-negative bacilli (GNB) group comprised patients whose microbiological culture revealed GNB other than *K. pneumoniae*, including *Escherichia coli*, *K. terrigena*, *Morganella morganii*, *Pseudomonas aeruginosa,* and *Proteus mirabilis*. The aerobic Gram-positive cocci (GPC) group included patients whose microbiological culture revealed GPC, including *Staphylococcus aureus* and viridans streptococci. The anaerobes group was composed of patients with anaerobic organisms in the microbiological culture, including *Fusobacterium necrogenes*, *F. varium*, and *F. nucleatum*. The mixed infection group included patients with more than 1 pathogen in the microbiological culture. Patients with polymicrobial infections were assigned to the mixed infection group. The mortality group included individuals who died within 6 weeks during the hospitalization from PPLA-related causes. The survivor group included those who did not die during the hospitalization. Abscesses were considered cryptogenic in origin when no causative lesions were apparent.

To analyze case characteristics and laboratory parameters of each group, descriptive statistics were calculated using SPSS version 10.0 for Windows (SPSS Inc., Chicago, IL, USA). Chi-square and Fisher’s exact tests were used for analysis. Results were considered significant if the *p*-value was <0.05.

## Results

One hundred fifty PPLA cases were diagnosed from January 1 to December 31, 2010. Sixteen cases were excluded because of inadequate data, leaving a total of 134 patients with documented PPLA. The incidence of PPLA in adults served by CCH was 275.4/100,000 patient-years in 2010. The male to female ratio was 0.3 (29.1). The mean age was 59.4 years (mean ± SD: 59.4 ± 16.3; median: 62.9; range: 9–88). The mean length of hospitalization was 14.6 days (mean ± SD: 14.2 ± 7.3; median: 15; range: 1–58). Clinically, 118 of 134 (88.1%) patients had fever, and 38 (26.7%) had low blood pressure (<90/60 mmHg) upon admission for PPLA. One hundred nine (85.2%) patients had right upper quarter pain at admission. The abscesses were located at the right liver lobe in 92 (68.7%), left lobe in 21, and both lobes in 7 cases. The liver abscess size ranged from 1 to more than 5 cm in diameter. One hundred twenty-seven (94.8%) patients had a single abscess, and 7 had multiple abscesses. Ninety-five (70.9%) out of 134 patients had abscesses that resulted from infection with *K. pneumoniae*. There were no statistically significant differences in the initial clinical presentations among the 134 patients, and symptoms at initial clinical presentations included fever, chills, abdominal pain, anorexia, malaise, nausea, emesis, body temperature higher than 38.3°C, right upper quadrant tenderness, Murphy's sign, hepatomegaly, disturbance of consciousness, and ascites. The initial ultrasonographic findings and the results of the initial ultrasound-guided aspiration biopsy are listed in Table 1.

**Table 1:** Initial ultrasonographic findings and initial aspiration among 134 pyogenic liver abscess patients

	***Klebsiella pneumonia *** **(n=95)**	**Non- *Klebsiella *** **GNB (n=31)**	**Aerobic GPC (n=5)**	**Anaerobes (n=1)**	**Mixed infection (n=2)**	**Total (n=134)**	**P-value**
Negative or equivocal findings of ultrasonographic examinations at first examination	8	1	1	0	1	11	1.000
Difficulty at initial aspiration	8	1	0	0	0	9	0.284
Gas formation	21	6	0	0	1	28	0.591
Multiple abscesses	2	4	1	0	0	7	0.022
Complications	5	3	1	0	1	10	0.001
Mortality	6	3	1	0	0	11	0.297

Notes: *abbreviation*: *GNB* Gram negative bacilli, *GPC* Gram positive cocci.

Every patient received treatment according to the PPLA treatment protocol (see Additional file 1). In every case, an empirical antibiotic treatment was started at the onset of the clinical signs of infection. Antibiotic therapies were changed based on the results of antibiotic susceptibility tests. Twenty-one (15.7%) patients developed complications, including endophthalmitis, PPLA rupture, and multiple organ failure. Of 134 patients, 6 died, leading to a crude mortality rate of 4.5% (6/134). The risk factors for mortality associated with PPLA included extra-hepatic complications, PPLA rupture, and multiple organ failure. One of 6 patients did not receive drainage because of bleeding tendency and ascites.

We compared the survivor and mortality groups to analyze the risk factors of the final outcome among the 134 patients with PPLA (Table 2). When compared with the survivor group, patients with mortality were male (26.6% vs. 83.3%, *p* < 0.001), malignancy (28.9% vs. 66. 7%, *p* < 0.001), respiratory distress (11.7% vs. 50%, *p* = 0.007), low blood pressure (29.7% vs. 33.3%, *p* = 0.001), jaundice (15.6% vs. 83.3%, *p* < 0.001), rupture of liver abscess (0% vs. 33.3%, *p* < 0.001), endophthalmitis (5.6% vs. 33.3%, *p* = 0.003), and multiple organ failure (3.1% vs. 100%, *p* < 0.001). In our study, no patients received liver transplantation, and none were diagnosed with HIV during the study period.

**Table 2:** Analysis of demographic data, clinical features, concomitant diseases and outcome among 134 patients with primary pyogenic liver abscess

	**Survivor**	**Mortality**	**Total**		**Univariate logistic regression analysis**		
	**N = 128 (%)**	**N = 6 (%)**	**N = 134(%)**	**p value**	**Odds ratio**	**95% confidence interval**	**P-value**
Demographic data							
Gender, male (%)	34 (26.6%)	5 (83.3%)	39 (29.1%)	<0.001	1.879	2.284–11.130	0.006
Age, mean ± SD^*1^ (years)	53.6 ± 18.9	67.5 ± 7.4	59.4 ± 16.3	0.705			
Length of hospitalization, mean ± SD (days)	10.2 ± 8.3	17.2 ± 6.3	14.2 ± 7.3	0.612			
Underlying diseases & co-morbidity							
Category C of disease severity on admission a^*2^, mean ± SD	15 (11.7%)	2 (33.3%)	17 (10.4%)	0.025			
Coexisting diseases, no. (%)							
Diabetes mellitus	84 (65.6%)	2 (33.3%)	86 (64.2%)	0.055			
Biliary disorders^*3^	44 (34.4%)	4 (66. 7%)	48 (35.8%)	0.055			
Alcoholism	45 (35.2%)	2 (33.3%)	47 (35.1%)	1.000			
Liver cirrhosis	46 (35.9%)	3 (50%)	49 (36.6%)	0.530			
Malignancy^*4^	37 (28.9%)	4 (66. 7%)	41 (30.6%)	<0.001	2.067	1.174–13.130	0.004
Uremia	49 (38.3%)	3 (50%)	52 (37.6%)	0.750			
Delay issue							
Patient delay (days), mean (range)	10 (1–124)	9 (1–91)		0.569			
Total delay (days), mean (range)	19 (1–124)	17 (1–91)		0.459			
Symptoms and signs on admission, no. (%)							
Fever/chills	118 (92.2%)	4 (66.7%)	122 (91.0%)	0.069			
Abdominal pain	117 (91.4%)	5 (83.3%)	122 (91.0%)	0.256			
Anorexia	12 (9.4%)	2 (33.3%)	14 (10.5%)	0.056			
Malaise	115 (89.9%)	5 (83.3%)	120 (89.6%)	0.322			
Nausea/emesis	114 (89.1%)	4 (66.7%)	118 (12.7%)	0.057			
Respiratory distress^*5^	15 (11.7%)	3 (50%)	18 (13.4%)	0.007	1.667	1.164–14.210	0.006
Signs on admission, no. (%)							
Body temperature > 38.3°C	111 (86.7%)	6 (100%)	117 (87.3%)	0.358			
Blood pressure < 90/60 mmHg	38 (29.7%)	2 (33.3%)	40 (29.8%)	0.001	2.167	2.104–13.150	0.003
Right upper quadrant tenderness	109 (85.2%)	3 (50%)	112 (83.6%)	0.701			
Jaundice	20 (15.6%)	5 (83.3%)	25 (18.7%)	<0.001	1.900	1.246–3.297	0.008
Murphy's sign^*6^	21 (16.4%)	1 (16.7%)	22 (16.4%)	0.869			
Hepatomegaly	22 (17.2%)	0 (0%)	22 (16.4%)	0.125			
Disturbance of consciousness	23 (17.8%)	1 (16.7%)	24 (17.9%)	0.980			
Ascites	24 (18.8%)	1 (16.7%)	25 (18.7%)	1.000			
Source of infection, no. (%)							
Biliary origin^*7^	32 (25%)	4 (66.7%)	36 (26.9%)	0.069			
Cryptogenic origin	33 (25.8%)	1 (16.7%)	34 (25.4%)	0.729			
Sonographic findings at first examination, no (%)							
Negative findings and equivocal	5 (83.3%)	1 (16.7%)	6 (4.5%)	0.077			
Single abscesss (77%)	101 (98.1%)	2 (1.9%)	103 (77%)	0.063			
Right lobe (72.3%)	95 (97.9%)	2 (2.1%)	97 (72.3%)	0.071			
Multiple abscesses (23%)	30 (96.8%)	1 (3.2%)	31(23%)	1.000			
Gas formation	27 (96.4%)	1 (3.6%)	28 (20.9%)	1.000			
Difficulty at initial aspiration	8 (88.9%)	1 (11.1%)	9 (6.7%)	0.161			
Microbiology				0.028			
*Klebsiella pneumonia*	93 (97.9%)	2 (2.1%)	95 (70.8%)				
Non- *Klebsiella* GNB	28 (90.3%)	3 (9.7% )	31 (23.1%)				
Aerobic GPC	4 (80%)	1 (20%)	5 (3.7%)				
Anaerobes	1 (100%)	0	1 (0.4%)				
Mixed infection	2 (100%)	0	2 (1.4%)				
Treatment							
Doctor delay of effective antibiotics therapy (days), mean (range)	8 (0–33)	4 (0–37)		0.101			
Prolonged doctor delay of effective antibiotics therapy (>8 days)	78 (61.5%)	2 (33.3%)		0.118			
Delay of aspiration/drainage (days), mean (range)	9 (0–33)	7 (0–27)		0.201			
Prolonged delay of aspiration/drainage therapy (>9 days)	78 (61.5%)	1 (16.7%)		0.008			
Complications, no. (%)							
Rupture of liver abscess	0 (0%)	2 (33.3%)	2 (1.5%)	<0.001	5.167	2.194–23.150	0.003
Endophthalmitis, related to PPLA	7 (5.6%)	2 (33.3%)	9 (6.7%)	0.003	2.167	1.234–13.140	0.005
Multiple organ failure, related to PPLA	4 (3.1%)	6 (100%)	10 (7.5%)	<0.001	3.067	1.184–15.150	0.001

P-value by Chi-square test or Fisher's exact test when appropriated.

*Abbreviation*: *GNB* Gram negative bacilli, *GPC* Gram positive cocci.

Notes:^*1^*SD* standard deviation, ^*2^Disease severity was categorized according to McCabe & Jackson criteria, and Category C meant the rapid fatal group; ^*3^Biliary disorders including biliary stone diseases (cholelithiasis, choledocholithiasis, or hepatolithiasis) and prior hepatobiliary; surgery; ^*4^Malignancy including lung cancer, pancreatic cancer, breast cancer, esophagus cancer, stomach cancer, colon cancer, lymphoma, prostate cancer, hematological malignancy, oral cancer, solid tumor and large intestines. Non-digestive system cancer; ^*5^Respiratory distress including shortness of breath, dyspnea, chest distress, hypoxemia (saturation of oxygen is less than 95%); ^*6^Murphy’s sign: deep inspiration or cough during subcostal palpation of RUQ producing increased pain and inspiratory arrest; ^*7^Biliary origin of liver abscess including suppurative cholangitis and acute cholecystitis.

According to the results of univariate logistic regression analysis, gender (OR = 1.185, 95% CI: 0.284–11.130, *p* = 0.006), malignancy (OR = 2.067, 95% CI: 1.174–13.130, *p* = 0.004), respiratory distress (OR = 1.667, 95% CI: 1.164–14.210, *p* = 0.006), low blood pressure (OR = 2.167, 95% CI: 2.104–13.150, *p* = 0.003), jaundice (OR = 1.9, 95% CI: 1.246–3.297, *p* = 0.008), rupture of liver abscess (OR = 5.167, 95% CI: 2.194–23.150, *p* = 0.003), endophthalmitis (OR = 2.167, 95% CI: 1.234–13.140, *p* = 0.005), and multiple organ failure (OR = 3.067, 95% CI: 1.184–15.150, *p* = 0.001) differed significantly between the mortality and survivor groups.

## Discussion

To the best of our knowledge, this is the first study to analyze the initial clinical presentations and initial ultrasonographic findings in patients with PPLA caused by different pathogens. Importantly, in 6 (7.5%) of 80 patients with bacteremia, infectious foci at the initial stage were not detected during the abdominal ultrasonographic examination (Table 1), but were revealed with abdominal CT. Lin, et al. showed that 38 (14.1%) of 268 PPLA patients had false-negative findings on ultrasonography at initial presentation [5]. Appropriate targeted antimicrobial treatments, combined with drainage of liver abscesses, have already increased PPLA survival rates [1,4]. Earlier diagnosis of PPLA could minimize sequelae and improve clinical outcomes [4]. Therefore, clinicians should pay special attention to bacteremic patients without obvious infectious foci. In particular, abdominal CT may be useful in patients at high risk of PPLA if initial results of abdominal ultrasonography are negative.

Although 9 of the 134 (6.7%) patients from this study did not undergo ultrasound-guided aspiration biopsy because of aspiration difficulties, their final clinical outcomes did not differ from those of the rest of the patients (Table 2). Evaluation of the final outcomes and duration of the treatment with antimicrobial agents revealed no significant differences between the patients without gas-forming and multiple abscesses (Table 1). In contrast to the results of Chou et al. [11], the current study and some previous studies [12,13] suggest that abscess multiloculation is not the single risk factor for PPLA mortality.

We compared the clinical presentations among different causative pathogens, including *K. pneumonia,* the main cause of liver abscesses in Taiwan with increasing infection prevalence [14]. There were no significant differences among the causative pathogens. In agreement with the previous reports from Taiwan, *K. pneumoniae* was the most predominant pathogen [15]. Sachdev et al. suggested that serotype K1 is an important factor of complicated endophthalmitis in *K. pneumonia-*caused PPLA [16]. We did not test the serotype of *K. pneumoniae* because of the lack of adequate equipment. In brief, there was no significant difference in *K. pneumonia*-related PPLA (97.9% vs. 2.1%, *p* = 0.297) between the survival and mortality groups in this study. The different of our results from previous studies [4,14,15] could be related to multiple factors, including initial broad-spectrum antibiotics prescription, and fewer K1 serotype of *K. pneumoniae* in Changhua County, and it’s needed further experiments to elucidate these points.

With the development of appropriate antimicrobial treatments and drainage methods for liver abscesses, PPLA survival rates have increased during this century [1,4]. In our study population, the crude mortality rate was 4.5% (6/134). Overall, the crude PPLA mortality rate has varied in the recent decade, with the average value being around 5% [7,16-18]. The mortality rates varied according to the differences in the geographic origins, study designs, study arms, and patient populations [4,17]. A literature review indicated that malignancy on presentation is an important risk factor for PPLA mortality [18-20], similar to our results. In our study, the risk factors associated with PPLA mortality included gender, jaundice, rupture of liver abscess, and multiple organ failure. Some of these factors have been described previously, including jaundice [21], rupture of liver abscess [21,22], and endophthalmitis [22]. Although the differences between genders could be related to testosterone levels [23], further experiments will be need to verify this. Multiple organ failure, initial low blood pressure, and initial respiratory distress are poor prognostic factors that result from higher disease severity, contributing to the higher mortality rates in the corresponding groups. In agreement with the results of Lin et al. [24], our study revealed an association between the prolonged delay of effective antibiotics and aspiration/drainage therapies and the risk of mortality. Although in the present study the time intervals between the presentation and initiation of effective antibiotics therapy varied within a wide range because of the non-specific and highly variable nature of PPLA presentations, we strongly recommend that an early, empirical, short-course, broad-spectrum antibiotics therapy is considered when persistent fever or unstable hemodynamics occur during the initial stages of the empirical therapy. The Lin’s report identified 6 independent risk factors predicting severe complications of *K. pneumonia*-related liver abscess: thrombocytopenia (<100,000/mm3), alkaline phosphatase > 300 U/L, gas formation in the abscess, APACHE III score > 40, use of cefazolin (instead of extended-spectrum cephalosporin), and delayed drainage [24,25].

Our study has several strengths. Most importantly, it represents an reliable reference for evaluating the initial clinical features of PPLA, providing valuable epidemiological information regarding the confirmed PPLA cases in central Taiwan. In addition, the prognostic factors, including the initial presentations, were analyzed. The limitations of this study include the retrospective cross-sectional design. The true prevalence of PPLA may be under-estimated because we collected only the cases with definitive diagnosis and positive microbiological findings in order to reduce potential confounding factors. Every case of polymicrobial liver abscess was considered a single case, with the predominant pathogen determined based on the results of microbiological evaluation. Similarly, only the results of liver abscess pus culture were taken into account if both blood and pus cultures were positive. Furthermore, since recurrent PPLA was counted as one case, the true incidence of PPLA is likely to be under-estimated. Finally, we did not calculate the time interval between definitive diagnosis and the initiation of effective antibiotic treatment.

## Conclusions

Because of early diagnosis and availability of more effective treatments, PPLA mortality is currently decreasing. Nevertheless, complications and mortality were still present in the current study. Although the initial presentations of PPLA were not significantly different among the groups with different causative pathogens, the disease was treated successfully in the majority of the patients. The mortality of the patients with PPLA was associated with: (1) male gender, (2) presence of malignancy, (3) initial respiratory distress, (4) initial low blood pressure, (5) jaundice, (6) rupture of liver abscess, (7) endophthalmitis, and (8) multiple organ failure. We strongly recommend using a disease severity score to determine the risk of mortality for each patient with PPLA. In order to prevent complications and reduce mortality, more attention must be paid to high-risk PPLA patients.

### Ethical approval

The study was approved by the institutional review board of Changhua Christian Hospital (CCH IRB No. 131116).

## Abbreviations

CCH: Changhua Christian hospital; CCHS: Changhua Christian Hospital System; CT: Computed tomography; GNB: Gram-negative bacilli; GPC: Gram-positive cocci; PPLA: Primary pyogenic liver abscess.

## Competing interests

The authors declare that they have no competing interests.

## Authors’ contributions

C-HC, S-SW, H-CC performed the majority of clinical services. C-HC coordinated this study and wrote the manuscript. Y-JC handled the statistical analysis. All authors read and approved the final manuscript.

## Pre-publication history

The pre-publication history for this paper can be accessed here:

http://www.biomedcentral.com/1471-230X/14/133/prepub

## Supplementary Material

Additional file 1 Treatment protocol for pyogenic liver abscesses. Click here for file
