# Supplementary material for: Initial presentations and final outcomes of primary pyogenic liver abscess: a cross-sectional study
Source: BMC Gastroenterol. 2014 Jul 28;14:133. doi: 10.1186/1471-230X-14-133 (PMC4115157; doi:10.1186/1471-230X-14-133)
Supplement: Additional file 1 — Treatment protocol for pyogenic liver abscesses. [file 1471-230X-14-133-S1.docx]

Treatment protocol for pyogenic liver abscesses

(1)Medical Care

An untreated liver abscess is nearly uniformly fatal due to complications that include sepsis, empyema, or peritonitis from rupture into the pleural or peritoneal spaces, and retroperitoneal extension. Treatment should include drainage, either percutaneous or surgical. Antibiotic therapy as a sole treatment modality is not routinely advocated, though it has been successful in a few reported cases. It may be the only alternative in patients too ill to undergo invasive procedures or in those with multiple abscesses not amenable to percutaneous or surgical drainage. In these instances, patients are likely to require many months of antimicrobial therapy with serial imaging and close monitoring for associated complications. Antimicrobial treatment is a common adjunct to percutaneous or surgical drainage.

(2)Surgical Care

Surgical drainage was the standard of care. With the refinement of image-guided techniques, percutaneous drainage and aspiration have become the standard of care.

Current indications for the surgical treatment of pyogenic liver abscess are for the treatment of underlying intra-abdominal processes, including signs of peritonitis; existence of a known abdominal surgical pathology (eg, diverticular abscess); failure of previous drainage attempts; and the presence of a complicated, multiloculated, thick-walled abscess with viscous pus. Shock with multisystem organ failure is a contraindication to surgery.A laparoscopic approach is also commonly used in select cases. This minimally invasive approach affords the opportunity to explore the entire abdomen and to significantly reduce patient morbidity. A growing literature is defining the optimal population for this mode of intervention. Postoperative complications are not uncommon and include recurrent pyogenic liver abscess, intra-abdominal abscess, hepatic or renal failure, and wound infection.

(3)Consultations

Interventional radiology

Obtain a consultation as soon as the diagnosis is considered to allow rapid collection of cavity fluid and the potential for early therapeutic drainage of abscess. General surgery Immediately seek a consultation with a general surgeon if the source of the abscess is a known underlying abdominal pathology or in cases with peritonitis. In cases undergoing percutaneous drainage, seek the involvement of a general surgeon if drainage of the abscess cavity is unsuccessful.

Gastroenterology

Gastroenterology involvement may be useful after successful drainage to evaluate for underlying gastrointestinal disease using colonoscopy or endoscopic retrograde cholangiopancreatography (ERCP).

Infectious disease specialist

Infectious disease consultation should be considered in complicated cases and when the involved pathogens are unusual or difficult to treat, such as in fungal abscesses.
